# The epidemiological investigation of co‐infection of major respiratory bacteria with pseudorabies virus in intensive pig farms in China

**DOI:** 10.1002/vms3.289

**Published:** 2020-06-24

**Authors:** Xuexiang Yu, Qi Sun, Xugang Ku, Dongxian He, Zhonghua Li, Ahmed H Ghonaim, Shengxian Fan, Qigai He

**Affiliations:** ^1^ State Key Laboratory of Agricultural Microbiology College of Veterinary Medicine Huazhong Agricultural University Wuhan China; ^2^ The Cooperative Innovation Center for Sustainable Pig Production Wuhan China; ^3^ Guangxi Agricultural Vocational College Nanning China; ^4^ Desert research center Cairo Egypt

**Keywords:** bacteria, mixed infection, pseudorabies virus, serotypes

## Abstract

Porcine respiratory disease complex (PRDC), a respiratory disease caused by a variety of factors, is one of the most common problems in the intensive pig farms. To investigate the mixed infection incidence of wild‐type pseudorabies virus (WT PRV) and respiratory bacteria, a total of 1,293 clinical samples were collected from pigs with typical respiratory signs from 14 different provinces of China from September 2016 to February 2018. The WT PRV was detected by ELISA targeting *gE* antibody while the bacteria were detected by bacterial isolation and serotyping by PCR. The results revealed that the detection rate of *A. pleuropneumoniae* and *B. bronchiseptica* infection associated with WT PRV infection were 6.30% and 15.99%, respectively, which were significantly higher than those without WT PRV infection (3.41% and 4.41%) at the farm level (*p* < .05). There were no significant differences in the detection rate of *H*. *parasuis*, *S. suis* or *P. multocida* between WT PRV positive and negative farms (*p > *.05). However, the detection rate of attenuated *H*. *parasuis* and *S. suis* strains were 68.19% and 64.75%, respectively, in WT PRV infected farms, which were significantly higher than those (41.56% and 52.25%) in WT PRV free farms (*p* < .05). The prevalent serotypes of *H*. *parasuis*‐5/12 and *S. suis*‐2 were also investigated by multiplex PCR. These results indicated that the presence of WT PRV increased the chance of bacterial infection and the number of pathogenic strains in the respiratory system of pigs. Therefore, the eradication of pseudorabies is an effective approach to prevent and control the bacterial respiratory diseases in the intensive pig farms in China.

## Graphical abstract

In this study, bacterial isolation and serotyping were performed from WT PRV negative and positive pigs, and then analysed for the impact of WT PRV infection on bacterial respiratory diseases in intensive pig farms in China. Explain the immunosuppressive effect of WT PRV at the clinical level. These results indicated that the WT PRV enhanced the possibility of bacterial infection and the bacterial load of pathogenic strains in the respiratory system of pigs.

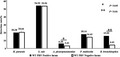

**Impacts**

- In this study, bacterial isolation and serotyping were performed from WT PRV negative and positive pigs, and then we analysed the impact of WT PRV infection on bacterial respiratory diseases in intensive pig farms in China.
- Explain the immunosuppressive effect of WT PRV at the clinical level.
- These results indicated that the WT PRV enhanced the possibility of bacterial infection and the bacterial load of pathogenic strains in the respiratory system of pigs.

## 1 INTRODUCTION

Porcine Respiratory Disease Complex (PRDC) is a general term for respiratory disease caused by mycoplasma, viruses, pathogenic bacteria, low quality feed, poor management and environmental stress (Kim, Chung, & Chae, 2003). The PRDC related pathogens are Porcine reproductive and respiratory syndrome virus (PRRSV), Pseudorabies Virus (PRV), Porcine circovirus type 2(PCV‐2), *Streptococcus suis* (*S. suis*), *Haemophilus parasuis* (*H. parasuis*), *Pasteurella multocida* (*P. multocida*)*, Bordetella bronchiseptica* (*B. bronchiseptica*)*, Actinobacillus pleuropneumoniae* (*A. pleuropneumoniae*) and etc (Opriessnig, Gimenez‐Lirola, & Halbur, 2011). Although each different pathogen can cause separate disease alone, simultaneous infection with two or more pathogens can often lead to more serious clinical symptoms and lesions (Allan et al., 2000; Brockmeier, Loving, & Nicholson, 2008; Carvalho, Segalés, & Pijoan, 1997; Chang et al., 2005; Opriessnig et al., 2011).

Traditionally, PRV is an etiological agent causing reproductive failure in sows, nervous disorder in nursery and growing pigs, respiratory problem in growing and finishing pigs. PRV can inhibit the synthesis of chemokines (Viejo‐Borbolla, Ana, Enrique, & Alcamí, 2009), the transcription of interferon (Brukman & Enquist, 2006), the expression of MHC I molecules by shutting off host protein synthesis (Mellencamp, O'Brien, & Stevenson, 1991), and subsequently cause immune‐ suppression (Chinsakchai & Molitor, 1992). It was noted that PRV infection could increase the severity of bacterial pneumonia (Opriessnig et al., 2011). Lesions, such as polyarthritis and fibrinous pericarditis, are more abundant and acute in pigs with mixed challenge exposure, compared with pigs infected only with *S. suis* (Iglesias, Pijoan, & Molitor, 1992); PRV infection can allow *H. parasuis* to proliferate in the lung by destroying the respiratory epithelial cells of pigs (Narita, Kawashima, Matsuura, Uchimura, & Miura, 1994). The clinical symptoms of *A. pleuropneumoniae* became more severe with concomitant infection with PRV (Sakano et al., 1993); PRV and *P. multocida* mixed infection also produce more severe pneumonia than *P. multocida* infection alone, and lead to a significant decrease in the average daily weight gain (Fuentes & Pijoan, 1987). However, since 2011, outbreaks of PRV caused by novel variant strains has been documented in lots of swine farms in China, causing serious economic losses to the swine industry. Therefore, it is urgent to understand the co‐infection status of PRV and main bacteria in PRDC in the pig farms.

In this study, bacterial isolation and serotyping were performed from WT PRV negative and positive pigs, and then we analysed the impact of WT PRV infection on bacterial respiratory diseases in intensive pig farms in China. It might pave the way to control bacterial diseases of the porcine respiratory system to be more precisely and efficiently.

## 2 MATERIALS AND METHODS

### 2.1 Sample collection

To assess the mixed infection of WT PRV and respiratory bacteria in intensive pig farms (≥ 1,000 pigs), a total of 1,293 clinical samples were collected from pigs with typical respiratory signs from 14 different provinces, such as Hubei, Henan, Hunan, Hebei and others from September 2016 to February 2018. The clinical samples were collected from suspected pigs and shifted to our diagnostic laboratory. The collected samples included nasal swabs (total of 574), lungs (total of 334), spleens (total of 105), joint fluids (total of 89), brains (total of 110), tracheal fluids (total of 81). Then, under complete sterile measures to avoid cross‐contamination, bacterial isolation was performed immediately. Blood samples were collected from the jugular vein of pigs and kept in 5 ml blood‐collecting tubes without anticoagulant. The research was approved by the Ethics Committee of the Faculty of Veterinary Medicine of the Huazhong Agricultural University. All procedures regarding animal care and testing were carried out according to the recommendation of Hubei provincial public service facilities.

### 2.2 Serological detection of *gE* antibody against WT PRV

Commercially available PRV/AD *gE* Ab ELISA kit with sensitivity and specificity 96.7% and 99.8%, respectively (IDEXX, USA) was used to detect *gE* antibody, which differentiates between vaccinated and infected pigs. In this study, a herd was considered to be a positive herd if at least one WT PRV positive sow was detected. Otherwise, the herd was considered to be a negative herd.

### 2.3 Culture conditions and identification methods

Five common bacterial pathogens, including *S. suis*, *H. parasuis*, *P. multocida, B. bronchiseptica* and *A. pleuropneumoniae*, were isolated and identified, during which Tryptic Soy Broth (TSB), Tryptic Soy Agar (TSA) (Difco Laboratories, Detroit, USA) were used. Then 10 μg/mL of nicotinamide adenine dinucleotide (NAD) and 5% (v/v) inactivated cattle serum (Zhejiang Tianhang Biotechnology, Zhejiang, China) were added for isolation of *H. parasuis* and *A. pleuropneumoniae*. All plates were incubated at 37°C for 24 to 48 hr. The strains were further identified by colony morphology, Gram‐staining characteristics and oxidase (Gram‐negative bacilli) or catalase tests. Phenotypic methods or standard biochemical procedures were used to identify suspected bacteria as *S. suis*, *H. parasuis*, *P. multocida, B. bronchiseptica* and *A. pleuropneumoniae* based on the previous studies. All isolated bacteria were kept at −80°C.

### 2.4 PCR primer sequences

According to sequences published in previous literatures, primers were synthesized by Sangon Biotech Co., Ltd (Shanghai). The primers for amplifying target genes of *S. suis*, *H. parasuis*, *P. multocida, B. bronchiseptica* and *A. pleuropneumoniae* are listed in Table 1.

**TABLE 1:** PCR primers used in this study

Bacteria	Gene	Primer	Sequence(5’−3’)	Size (bp)	References
*S. suis*	16S rRNA	Forward Reverse	CAGTATTTACCGCATGGTAGAT GTAAGATACCGTCAAGTGAGAA	294	Cheung et al., (2008)
*A. pleuropneumoniae*	*ap x*IV	Forward Reverse	TGGCACTGACGGTGATGA GGCCATCGACTCAACCAT	377	Gram, Ahrens, Andreasen, and Nielsen (2000)
*H. parasuis*	16S rRNA	Forward Reverse	GTGATGAGGAAGGGTGGTGT GGCTCGTCACCCTCTGT	821	Oliveira, Galina, and Pijoan, (2001)
*B. bronchiseptica*	*f la*	Forward Reverse	GCTCCCAAGAGAGAAAGGCT GGTGGCGCCTGCCCTATC	235	Hozbor, Fouque, and Guiso, (1999)
*P. multocida*	*kmt 1*	Forward Reverse	ATCCGCTATTACCCAGTGG GCTGTAAACGAACTCGCCAC	457	Nagai, Someno, and Yagihashi, (1994)

### 2.5 Serotype identification of *S. suis* and *H. parasuis*

According to the reports, *S. suis* can be classified into 33 serotypes based on the difference of capsular polysaccharide (Liu et al., 2013), and *H. parasuis* can be classified into 14 serotypes based on the difference of capsular loci (Howell et al., 2015). *S. suis* and *H. parasuis* strains were randomly chosen for further serotyping by typing PCR according to the previously described methods.

### 2.6 Statistical analysis

At the farm level and the individual sample level, all research data were analysed to identify the statistical differences of bacterial respiratory diseases between the WT PRV free or positive farms. To avoid the confusion from the presence of maternal derived *gE* antibody, only data from breeding and fattening pigs were used.

Statistical analyses were undertaken with SAS version 9.0 (SAS Institute Inc.). Univariate association between variables and isolation rates of different bacteria were determined by using univariate ordinary logistic regression analysis and Chi‐square test. *p* < .05 and *p* < .01 were considered to be significant and highly significant, respectively.

## 3 RESULTS AND ANALYSIS

### 3.1 Bacterial test results of various samples

The results showed that, among the tested bacteria, the detection rate of *S. suis* was the highest in all types of samples, and for *S. suis*, the nasal swabs were the most suitable samples with the highest detection rate. The detection rate of *H. parasuis*, *P. multocida, B. bronchiseptica* and *A. pleuropneumoniae* was high in tracheal fluid samples. It can be noticed that the highest rate of respiratory bacteria could be observed in the tracheal fluid samples while the lowest rate in the joint fluid samples. Hence, the most suitable sample for respiratory bacterial isolation is the tracheal fluid samples. The detailed bacterial detection rates are shown in Table 2.

**TABLE 2:** Detection of bacteria in different types of samples

	Nasal swabs	Lungs	Spleens	Brains	Joint fluids	Tracheal fluids	Total
*H. parasuis*	23.00% (132/574)	20.36% (68/334)	9.52% (10/105)	10.00% (11/110)	8.99% (8/89)	43.21% (35/81)	20.42% (264/1293)
*S. suis*	69.86% (401/574)	44.31% (148/334)	42.86% (45/105)	40.00% (45/110)	20.22% (18/89)	56.79% (46/81)	54.29% (702/1293)
*A. pleuropneumoniae*	4.70% (27/574)	6.59% (22/334)	6.67% (7/105)	0.91% (1/110)	1.12% (1/89)	11.11% (9/81)	5.18% (67/1293)
*P. multocida*	16.38% (94/574)	25.45% (85/334)	7.62% (8/105)	4.55% (5/110)	2.25% (2/89)	27.16% (22/81)	16.71% (216/1293)
*B. bronchiseptica*	15.16% (87/574)	12.57% (42/334)	4.76% (5/105)	1.82% (2/110)	0% (0/89)	16.05% (13/81)	11.52% (149/1293)
Total	129.09% (741/574)	109.28% (365/334)	71.43% (75/105)	57.27% (63/110)	32.58% (29/89)	154.32% (125/81)	108.12% (1398/1293)

### 3.2 WT PRV *gE* antibody test results

Together with tissue samples, a total of 1,293 serum samples from 94 intensive pig farms were tested for presence of WT PRV infection by *gE*‐ELISA, of which 499 and 794 samples were from 45 and 49 WT PRV negative or positive pig farms, as shown in Table 3. The detection rates of *gE* antibody were further categorized corresponding to the growth stage of pigs from which sera were collected. In this study, only fattening and breeding pigs were selected to eliminate the effect of maternal derived antibodies,

**TABLE 3:** Number of samples at different growth stages in WT PRV negative and positive farms

Background	Growth stage				
	Piglets	Nursery	Fattening	Sow	Total
WT PRV positive farms	60.00% (30/50)	77.84% (260/334)	69.01% (147/213)	51.27% (101/197)	67.76% (538/794)
WT PRV negative farms	0% (0/50)	0% (0/343)	0% (0/103)	0% (0/3)	0% (0/499)
Total	30.00% (30/100)	38.40% (260/677)	46.52% (147/316)	50.50% (101/200)	41.61% (538/1293)

### 3.3 Detection rate of respiratory bacteria at WT PRV negative and positive farms "at the farm level"

**FIGURE 1:**
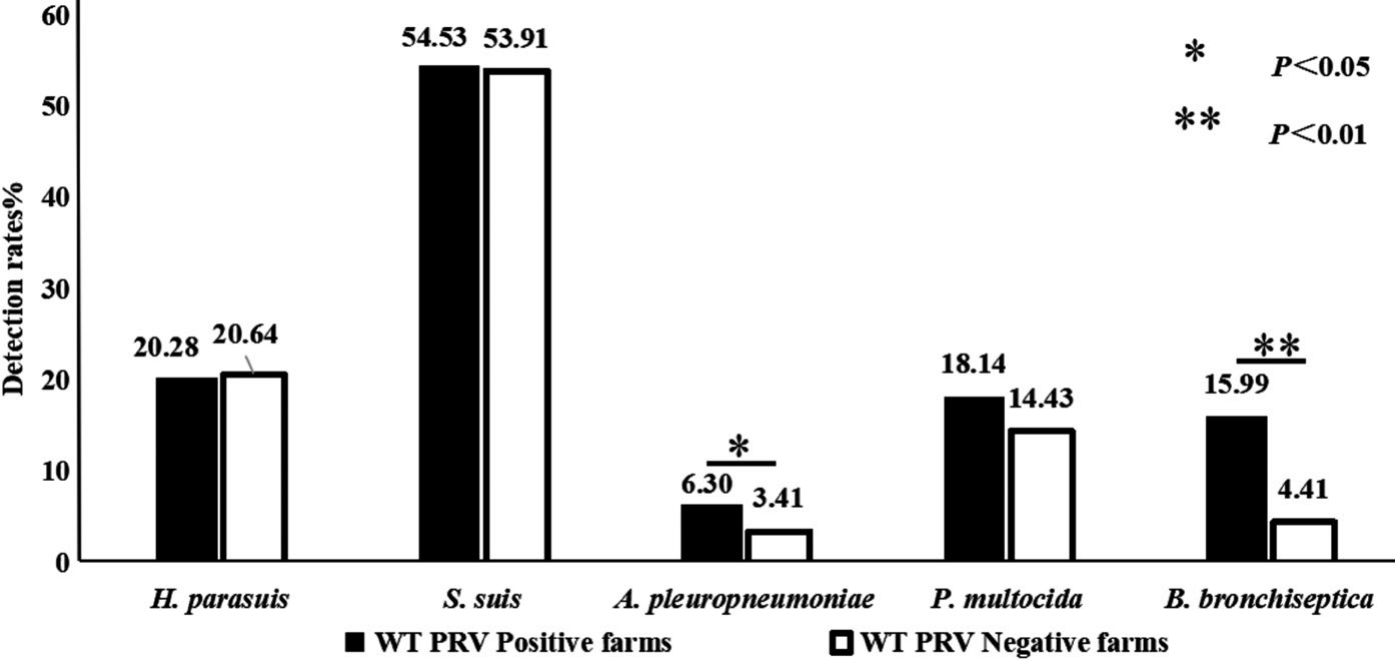
Detection rate of bacteria in WT PRV negative and positive fields

To analyse the difference in bacterial respiratory diseases between the WT PRV positive and negative farms, the detection rate of different respiratory bacteria was listed and compared (Figure 1).

There was no significant differences in the detection rate of *H. parasuis*, *S. suis* and *P. multocida* between WT PRV negative and positive farms. However, the detection rate of *B*. *bronchiseptica* and *A. pleuropneumoniae* were significantly higher in WT PRV positive farms than in PRV negative farms (*p* < .01).

### 3.4 Detection rate of respiratory bacterial infection with and without co‐infection with WT PRV "at the individual level"

**FIGURE 2:**
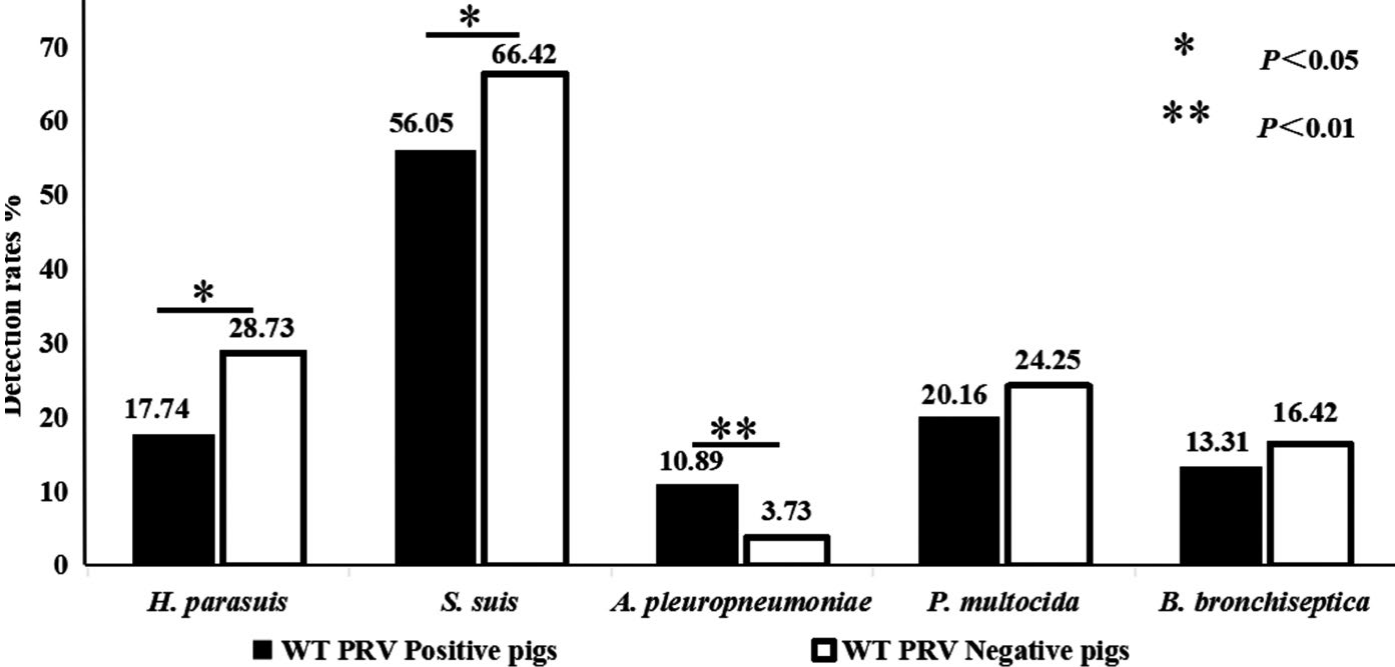
Detection rate of bacteria in WT PRV infected or free pigs

To illustrate the relationship among five bacterial infections either with or without PRV infection at the individual pig level, 516 samples from fattening and breeding pigs were collected where 268 and 248 samples were from WT PRV antibody negative or positive pigs. The detailed bacterial detection rates are shown in Figure 2.

Higher isolation rates of both *H.parasuis and S.suis* are found in WT PRV positive farms than in WT PRV negative pigs, on the contrary, lower isolation rate of *A.pleuropenumoniae* is found in WT PRV negative farms (3.73%) than in WT PRV positive pigs (10.89%). Significant differences were noticed in the infection rate of *H. parasuis*, *S. suis* and *A. pleuropneumoniae* between WT PRV positive and negative pigs. However, there were no significant differences in the detection rates of *P. multocida* and *B*. *bronchiseptica* in either WT PRV positive or negative pigs. Surprisingly, the infection rate of *H. parasuis* and *S. suis* in WT PRV infected pigs were significantly lower than those in pigs without WT PRV infection. Whereas, the infection rate of *A. pleuropneumoniae* was significantly higher in WT PRV infected pigs than in WT PRV free pigs.

### 3.5 Distribution of *H. parasuis* serotypes in WT PRV positive and negative pigs

Totally, 121 *H. parasuis* were identified from samples from finishing and breeding pigs, of which 44 and 77 were isolated from WT PRV positive and negative pigs, respectively. Serotypes 4 and 5 of *H. parasuis* were the main serotypes upon all conditions (Table. 4).

**TABLE 4:** Detection rate of *H. parasuis* serotypes in WT PRV positive or negative pigs

	WT PRV positive pigs	WT PRV negative pigs	Total
1	6.82% (3/44)	15.58% (12/77)	12.39% (15/121)
2	9.09% (4/44)	6.49% (5/77)	7.44% (9/121)
3	4.55% (2/44)	0% (0/77)	1.65% (2/121)
4	22.73% (10/44)	22.08% (17/77)	22.32% (27/121)
5/12	20.45% (9/44)	27.27% (21/77)	24.79% (30/121)
6	0% (0/44)	0% (0/77)	0% (0/121)
7	2.27% (1/44)	2.60% (2/77)	2.48% (3/121)
8	0% (0/44)	0% (0/77)	0% (0/121)
9	9.09% (4/44)	1.30% (1/77)	4.13% (5/121)
10	0% (0/44)	5.19% (4/77)	3.30% (4/121)
11	0% (0/44)	0% (0/77)	0% (0/121)
13	4.55% (2/44)	9.09% (7/77)	7.44% (9/121)
14	0.00% (0/44)	1.30% (1/77)	0.83% (1/121)
15	13.64% (6/44)	3.90% (3/77)	7.44% (9/121)
Nontypeable	6.82% (3/44)	5.19% (4/77)	5.78% (7/121)
Virulent strains	31.82% (14/44)	58.43% (45/77)	48.75% (59/121)
Avirulent strains	68.19% (30/44)	41.56% (32/77)	51.24% (62/121)
Total	100% (44/44)	100% (77/77)	100% (121/121)

Theoretically, the *H. parasuis* serotypes 1, 5, 10, 12, 13 and 14 are regarded as virulent strains. As a result, the detection rate of avirulent strains of *H. parasuis* was 41.56% in PRV free pigs, which was significantly lower than that of in WT PRV infected pigs (68.19%) (*p* < .01).

### 3.6 The distribution of *S. suis* serotypes in WT PRV positive or negative pigs

Totally, 317 *S. suis* were identified from the samples from finishing and breeding pigs. From them, 139 and 178 strains were isolated from WT PRV positive and negative pigs, respectively.

As shown in Table 5, the *S. suis* serotypes 1, 2, 7 and 9 were regarded as virulent serotypes. The detection rate of avirulent strains was 52.25% in PRV non‐infected pigs, which was also significantly lower than that in PRV infected pigs (64.75%) (*p* < .05).

**TABLE 5:** Detection rate of *S. suis* serotypes in WT PRV positive and negative pigs

	WT PRV positive pigs	WT PRV negative pigs	Total
1	3.60% (5/139)	8.99% (16/178)	6.62% (21/317)
2	17.27% (24/139)	21.91% (39/178)	19.87% (63/317)
3	10.07% (14/139)	5.06% (9/178)	7.26% (23/317)
4	0% (0/139)	0% (0/178)	0% (0/317)
5	2.88% (6/139)	3.37% (6/178)	3.15% (10/317)
6	2.16% (3/139)	1.69% (3/178)	1.89% (6/317)
7	6.47% (9/139)	7.30% (13/178)	6.94% (22/317)
8	15.83% (22/139)	8.99% (16/178)	11.99% (38/317)
9	7.91% (11/139)	9.55% (17/178)	8.83% (28/317)
10	0.72% (1/139)	1.12% (2/178)	0.95% (3/317)
Nontypeable	33.09% (46/139)	31.46% (56/178)	32.18% (102/317)
Virulent strains	35.25% (49/139)	47.75% (85/178)	42.27% (134/317)
Avirulent strains	64.75% (90/139)	52.25% (93/178)	57.73% (183/317)
Total	100% (139/139)	100% (178/178)	100% (317/317)

## 4 DISCUSSION

### 4.1 Detection rate of bacterial infection associated with or without WT PRV

It has been reported that nasal swabs and other samples can be used to detect respiratory diseases associated‐bacteria in pigs (Correa‐Fiz, Fraile, & Aragon, 2016; Garch, de Jong, & Simjee, 2016; Loera MuroIt, Avelar‐González, Loera‐Muro, Jacques, & Guerrero‐Barrera, 2013; Macinnes, Gottschalk, Lone, Metcalf, & Friendship, 2008). The results in this study showed that all of the five tested bacteria have higher detection rate in both nasal swabs and tracheal fluid samples than that in joint fluid. However, the nasal swab samples might contain more environmental bacteria, so the tracheal fluid sample is the best sample to isolate respiratory bacteria. The common bacteria as *S. suis*, *H. parasuis*, *P. multocida, B. bronchiseptica* and *A. pleuropneumoniae* were focused because these bacteria are at higher risk factors causing porcine bacterial respiratory diseases.

Maternally derived antibodies, such as *gE* antibody, in the serum of unvaccinated piglets born to the sows immunized with PRV vaccine are serological positive till about 10 to 11‐weeks old (Malgorzata, Markowska, & Pejsak, 2010) and will lead to the difficulty in differentiation between infection and vaccination, so, in this study, only fattening and breeding pigs were chosen for discussion of the potential effect of WT PRV infection on bacterial infection. However, the presence of PRV‐*gE* antibodies only indicated that pigs may be previously infected with WT PRV, not the natural status of simultaneous existence of pathogens. Therefore, co‐infection model under laboratory conditions is needed to confirm the effect of WT PRV infection on secondary infection of respiratory problem‐related bacteria. It was noted that WT PRV infection under laboratory conditions could increase the severity of swine pneumonia that were caused by singular pathogen, for example *S. suis*, *H. parasuis*, *A. pleuropneumoniae*, *P. multocida* and other bacteria (Fuentes & Pijoan, 1987; Iglesias et al., 1992; Naritaet al., 1994; Opriessnig et al., 2011; Sakano et al., 1993). However, there are few clinical data on secondary infection of respiratory disease‐related bacteria in individual level and herd level upon WT PRV infection. This study demonstrated that the detection rates of *A. pleuropneumoniae* and *B*. *bronchiseptica* in WT PRV infected pig farms were significantly higher than those in the WT PRV free pig farms, indicating that, in the WT PRV positive pig farms, more attention should be paid to secondary bacterial infection especially the avirulent strains of *H. parasuis and S. suis*.

### 4.2 The prevalent serotyes of respiratory disease‐related bacteria

In this study, the prevalent serotype of *S. suis* and *H. parasuis* were determined via serotyping PCR. The prevalent serotypes of *S.suis* between 2016 and 2018 were serotypes 1,2,7,9 in PRV positive and negative samples which are consistent with other descriptions (Zhang et al. 2019), but the isolation rate of these virulent serotypes (47.47%) were higher in WT PRV negative pigs than those (35.25%) in WT PRV positive pigs. The prevalent serotypes of *H.parasuis* in PRV positive and negative pigs were 1,5/12,13 (31.82%) and 1,5/12,10,13 and 14 (58.43%), respectively. The reason why the WT PRV negative pigs are more susceptible to *H. parasuis* infection needs further research. Notably, there is also high proportion of isolation rate of non‐typeable serotypes in both *S. suis* and *H. parasuis*.

Traditionally, the viral infection may plan a key role for secondary infection. Through serotyping, it was found that there was no difference in detection rates for virulent serotypes of the tested bacteria from samples collected from both WT PRV positive or negative samples or farms, while there is a difference among avirulent serotypes. This implies that the high virulent strains could alone infect pigs and lead to the economic losses independent of WT PRV infection. So, the specific prevention of these bacterial infections is via vaccination, medication and management. For the prevention of avirulent bacterial strain, due to the immunosuppression caused by WT PRV, the herd vaccination with commercially available vaccine in combination with depopulation may be beneficial for prevention of both PR and bacteria‐related respiratory problem.

### 4.3 The need for eradication of WT PRV

Nowadays, many swine farms, especially commercial ones, have not paid enough attention for the elimination of WT PRV. However, the fact that WT PRV infection could increase the chance of invasion of bacterial pathogens was clinically verified. Upon being infected with WT PRV, the pigs are more likely to be subsequently infected with virulent bacteria. In addition, PRV infection also increases the severity of bacterial pneumonia (Jeffrey, Locke, Alejandro, Kent, & Grego, 2012). It has been reported that when *S. suis* infected pigs are re‐infected with PRRSV, pigs would suffer from more serious damage (Xu et al., 2010). Hence, when bacterial infection of the respiratory system is accompanied with PRV infection, this can also lead to more serious results than PRV alone, resulting in serious negative effect on the pig production performance. The results in this study further emphasize that the eradication of WT PRV may reduce the possibility of secondary infection of bacteria, aiming to prevent the occurrence of the porcine respiratory diseases. Therefore, it is necessary to eradicate WT PRV in positive swine farms.

### 4.4 The level of biosecurity and husbandry management

Although this study only considers the relationship between pseudorabies and bacteria, CSF, PRRS and PCV2 also cause immune‐suppression and may induce similar results. It has been reported that PRRSV accelerates *S. suis* or *H. parasuis* infection in vivo and in vitro, and also causes more severe respiratory symptoms (Yu et al., 2012; Huong et al., 2016; Li et al., 2017, 2018) among pigs with clinical symptoms of respiratory diseases, the highest percentage of PCV2 infection was with P. multocida in all cases (Kim et al., 2003), pigs infected with PCV2 and PRRSV suffer from severe immune‐suppression, so it's easy for occurrence of secondary bacterial infection(Chang, Peng, Chang, Chaung, & Chung, 2008; Opriessnig et al., 2011; Wang et al., 2019). Also, the poor biosecurity and husbandry can cause more bacterial infection. Moreover, it can also cause outbreaks such as PR, CSF, PRRS and PCV2. Therefore, for better control of bacterial respiratory diseases, the first aim is to improve the level of biosecurity and husbandry management. Then carrying out the prevention and control of PR, CSF, PRRS, PCV2 and bacterial respiratory diseases can achieve better results in pig farms.

## 5 CONCLUSION

The prevalent serotypes of *H*. *parasuis* and *S. suis* were *H*. *parasuis*‐5/12 and *S. suis*‐2, accounting for 24.79% and 19.87%, respectively. When pigs were infected with WT PRV, it's more likely to enhance secondary infection by *S. suis* and *H. parasuis* avirulent strains, *A. pleuropneumoniae* and *B*. *bronchiseptica*. So, PRV‐positive farms should pay more attention to prevent secondary bacterial infection. Hence, WT PRV positive farms should eradicate WT PRV and improve the level of biosecurity and husbandry management.

## CONFLICTS OF INTEREST

We have no other conflicts to declare.

## AUTHOR CONTRIBUTION

**Xuexiang Yu:** Conceptualization; Data curation; Formal analysis; Investigation; Methodology; Software; Writing‐original draft. **Qi Sun:** Data curation; Investigation; Methodology; Writing‐review & editing. **Xugang Ku:** Data curation; Methodology; Resources; Supervision; Validation. **Dongxian He:** Investigation; Methodology; Supervision. **Zhonghua Li:** Investigation; Validation; Visualization; Writing‐review & editing. **Ahmed H Ghonaim:** Visualization; Writing‐review & editing. **Shengxian Fan:** Resources; Supervision; Validation; Visualization. **Qigai He:** Conceptualization; Funding acquisition; Project administration; Resources.
